# Selective gene silencing by viral delivery of short hairpin RNA

**DOI:** 10.1186/1743-422X-7-248

**Published:** 2010-09-21

**Authors:** Katja Sliva, Barbara S Schnierle

**Affiliations:** 1 Paul-Ehrlich-Institute, Paul-Ehrlich-Str. 51-59, 63225 Langen, Germany

## Abstract

RNA interference (RNAi) technology has not only become a powerful tool for functional genomics, but also allows rapid drug target discovery and *in vitro *validation of these targets in cell culture. Furthermore, RNAi represents a promising novel therapeutic option for treating human diseases, in particular cancer. Selective gene silencing by RNAi can be achieved essentially by two nucleic acid based methods: i) cytoplasmic delivery of short double-stranded (ds) interfering RNA oligonucleotides (siRNA), where the gene silencing effect is only transient in nature, and possibly not suitable for all applications; or ii) nuclear delivery of gene expression cassettes that express short hairpin RNA (shRNA), which are processed like endogenous interfering RNA and lead to stable gene down-regulation. Both processes involve the use of nucleic acid based drugs, which are highly charged and do not cross cell membranes by free diffusion. Therefore, *in vivo *delivery of RNAi therapeutics must use technology that enables the RNAi therapeutic to traverse biological membrane barriers *in vivo*. Viruses and the vectors derived from them carry out precisely this task and have become a major delivery system for shRNA. Here, we summarize and compare different currently used viral delivery systems, give examples of *in vivo *applications, and indicate trends for new developments, such as replicating viruses for shRNA delivery to cancer cells.

## Introduction

The human genome project not only unraveled the human genetic code, but spin-off technical improvements also inspired genome sequencing of a multitude of other organisms. However, since sequence data alone are not sufficient to identify gene function, gene knock-out or knock-in strategies have to replenish the results in order to analyze the resulting phenotypic changes defining gene functions.

Knowledge about the *in vivo *phenotype after knocking out gene products is a prerequisite to assess the therapeutic potential of inhibitors against specific targets, so in drug development knock-out animal models have become very important. However, generating transgenic animals is still very labor and cost intense. Alternatively, selective silencing can be achieved by exploiting the RNA interference (RNAi) machinery of the host cell.

Since its discovery by Fire and Mello [1] in *C. elegans *in 1998, which gained them the Nobel prize in 2006, and by Tuschl *et al. *[2] in mammalian cells in 2001, RNAi was quickly adopted by the research community as a versatile tool with a wide range of applications, from reverse genetics to high throughput screening of drug targets. The key therapeutic advantage of using RNAi lies in its ability to specifically and potently knock-down the expression of disease-causing genes of known sequence.

Although RNAi is in comparison to knock-out strategies, able to only knock-down the gene expression, simple *in vivo *inhibition of single gene products by RNAi yields phenotypes that are comparable to classical knock-out animals used for therapeutic target identification or validation. Furthermore, basic research benefits from *in vivo *RNAi as this strategy can be changed dependent on the desired outcome. For example, conditional gene knock-outs utilizing inducible promoters can be used to unravel molecular pathways and investigate functional genomics.

RNAi is a basic pathway in eukaryotic cells. In contrast to activating cascades in cells upon exposure to long double stranded RNA, leading to non-specific RNA degradation, RNAi is mediated by short RNA duplexes hitchhiking a cellular pathway that silences genes in a sequence-specific manner at the mRNA level. Perfectly complementary dsRNA (short hairpin RNA, shRNA) is chopped up by Dicer, a ribonuclease III (RNase III) family member, into small interfering RNA (siRNA) duplexes 21-23 nt in length with symmetric 2-3 nucleotide (nt) 3' overhangs [3]. The use of siRNA duplexes is often accompanied by off-target effects, which can be avoided or reduced by adding backbone modifications to the duplexes to alter key thermodynamic and binding properties [4-7]. DICER-chopped duplexes are incorporated into a protein complex called the RNA-induced silencing complex (RISC) and subsequently unwound by the multi-functional protein Argonaut 2, contained within RISC. The activated RISC, which contains the antisense strand (or guide strand) of the siRNA, is then thought to direct the siRNA to the target mRNA with identical sequence. This leads to degradation of the target mRNA. The activated RISC complex can then move on to destroy additional mRNA targets, which further propagates gene silencing [8,9]. This feature of the RNAi mechanism induced by synthetic siRNA provides a knock-down effect for up to seven days in rapidly dividing cells and for several weeks in resting cells [10,11]. Thus, RNAi provides a simple, inexpensive and selective method for gene inhibition with a high success rate [12].

Eukaryotes produce various types of small RNAs that function in diverse pathways [3,13-15]. Since in some species the active forms of small RNAs are often indistinguishable biochemically or functionally, they are conventionally grouped into two classes based on their origins and their biogenesis: microRNAs (miRNAs) and small interfering RNAs (siRNAs). MiRNAs are generated from the dsRNA region of the hairpin-shaped precursors, whereas siRNAs are derived from long double-stranded RNAs (dsRNAs) [16]. MiRNAs are transcribed as primary miRNA transcripts (pri-miRNAs) which are then processed within the nucleus by a complex consisting of RNAse III enzyme Drosha and the double-stranded RNA-binding protein DGCR 8 into pre-miRNAs. These are exported from the nucleus into the cytoplasm by exportin-5. In the cytoplasm the pre-miRNA enters the same pathway as the above mentioned siRNA. At the end both miRNAs and siRNAs bind to mRNA and induce mRNA cleavage, translational repression, and cleavage-independent mRNA decay [17]. While miRNAs predominantly induce translational repression due to imperfect pairing to their target mRNA, siRNAs often form a perfect duplex with their target and therefore direct the cleavage of the target mRNAs at the site of complementarity.

**Figure 1:**
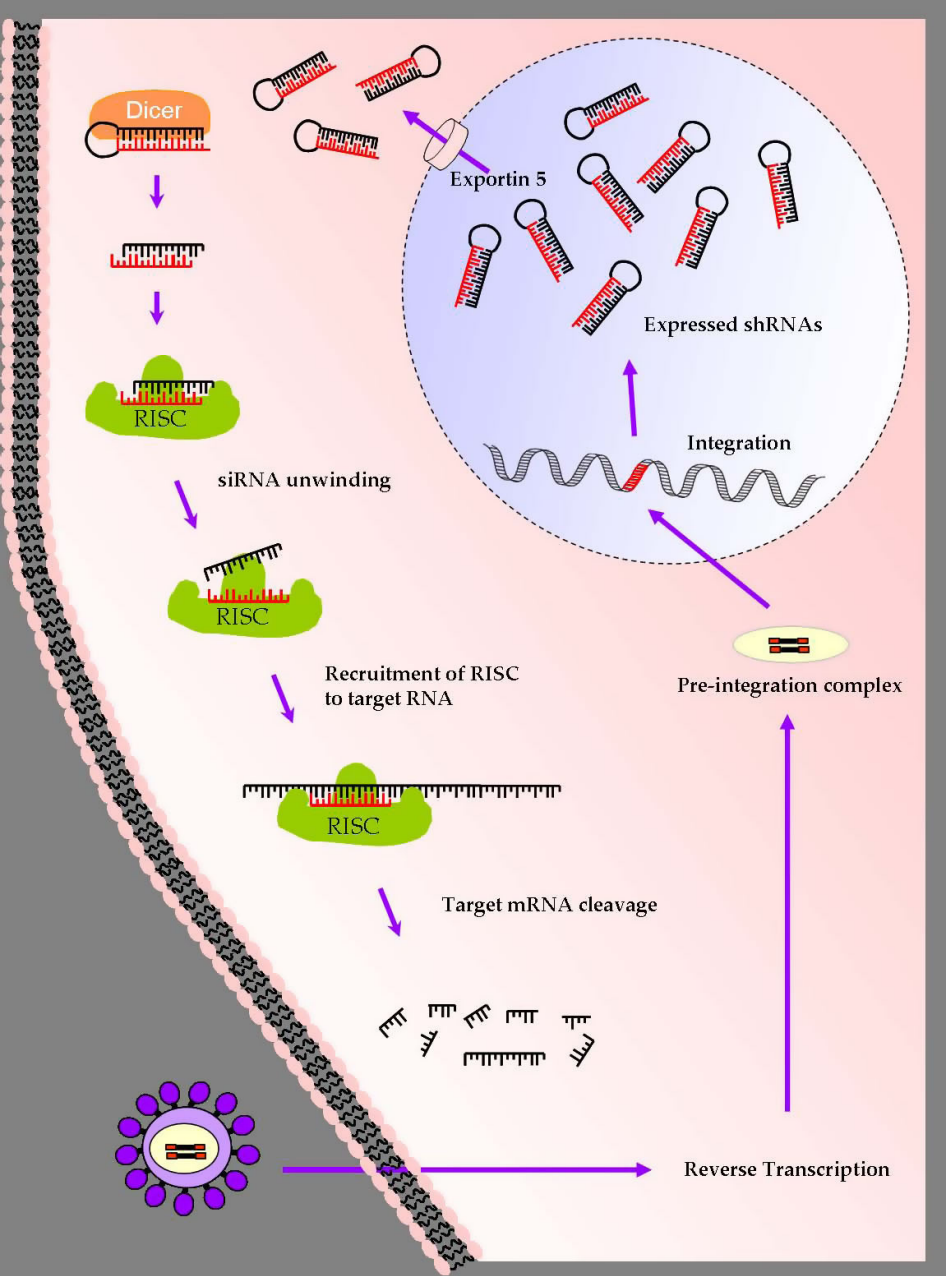
**Schematic overview of the mechanism of RNA silencing in the host cell that leads to transcriptional silencing after retroviral delivery of sh/miRNA**. Retroviruses (or vectors) deliver therapeutic shRNA-expressing transgenes that integrate into the genome of the host cell and lead to stable shRNA expression. Expressed shRNAs require the activity of endogenous Exportin 5 for nuclear export [129]. Several proteins are recruited and form a dimer with Dicer which receives and subsequently cleaves the dsRNA generating duplex siRNAs with 2 nt 3' overhangs. These siRNAs activate the RNA-induced silencing complex (RISC) which unwinds the RNA and recruits only the guiding strand to target mRNA which is subsequently cleaved and degraded. The figure is schematic, and the Dicer and RISC complexes can vary dependent on cellular process.

Figure 1 schematically summarizes the RNAi machinery of the host cell.

## Potential therapeutic targets for RNA interference

RNAi-based therapy for human cancer is one of the most rapidly progressing applications for virally delivered shRNA [18-20]. Theoretically, when using appropriately designed siRNA, the RNAi machinery can be exploited to silence almost any gene in the genome. Indeed, it has already been reported that synthetic siRNAs are capable of knocking down targets in various diseases *in vivo *[21-23]. Experimentally tested, effective targets are genes involved in cancer-associated cellular pathways, either oncogenes, particularly fusion oncogenes due to their unique link with certain tumor cells, or anti-apoptotic genes. In addition, genes that play a role in tumor-host interactions, such as factors involved in angiogenesis or innate immunity, and those that mediate resistance to chemo- or radiotherapy are targets for interference [24,25]. For instance, cancer disease such as ovarian cancer [26] and bone cancer [27] are currently being investigated and successfully treated with siRNAs *in vivo*.

A further interesting therapy field is the area of miRNA-caused malignancies. The direct effects of miRNA, which are believed to regulate as many as one-third of all human gene transcripts (or messenger RNAs), are implicated in many human diseases. Using gene therapy to manipulate miRNA levels represents an attractive new approach for controlling gene expression and identifying targeted and effective therapeutics [28]. An important role for miRNAs in cancer pathogenesis has emerged over the last few years, and many reports reveal numerous examples linking dysregulated expression of miRNAs to cancer [29,30]. Recent results demonstrated that expression of a single miRNA *in vivo *can reverse disease progression in a liver cancer model [31]. This opened up a whole new replacement therapy field for cancer treatment using RNAi.

Human pathogenic viruses are also excellent targets for RNAi, because, as exogenous sequences, they are unique in the host, which minimizes off-target side effects due to the treatment. Here, the strategy is to target essential viral genes to prevent viral proliferation. However, one has to keep in mind that some viruses have acquired the ability to counteract anti-viral RNAi. Examples of successful RNAi approaches to combat human pathogenic viruses include targeting Hepatitis B Virus (HBV) [32,33], Human Papilloma Virus [34], Severe Acute Respiratory Syndrome (SARS) Coronavirus [35], and Respiratory Syncytial Virus (RSV) infections [36].

Other therapeutically relevant fields are metabolic diseases, cardiac disorders, human neurodegenerative diseases and inherited genetic diseases. A recently published study showed successful siRNA targeting of PCSK9, a member of the mammalian serine protein convertase family, that typically functions in proteolytic processing and maturation of secretory proteins and was the first family member to be implicated in a dominantly inherited form of hypercholesterolemia [37]. Targeting PCSK9 with siRNA lowered plasma cholesterol and hence offers an auspicious therapeutic approach to controlling this disease. Clinical trials for coronary artery disease are also underway, using an RNA therapeutic agent aimed at silencing one of the genes (c-myc) responsible for causing arteries to reclose after stent insertion (restenosis) [38]. Another study showed almost completely resolved liver fibrosis and prolonged survival *in vivo *in rats following treatment with synthetic and modified siRNAs. The efficacy highlighted a new therapeutic potential for reversing human liver cirrhosis [39]. Table 1 provides a rough overview of current clinical trials for siRNA therapeutics.

**Table 1:** Current Clinical Trials for siRNA Therapeutics

Disease	Mode of administration	Status	Company
Age-related macular degeneration (AMD)	Topical	Phase II	Allergan

Respiratory syncytial virus (RSV)	Local/direct	Phase II	Alnylam

Liver cancer (HCC and others)	Systemic	Phase I	Alnylam

Hepatitis B Virus (HBV)	Systemic	Phase I	Nucleonics

Solid tumors	Systemic/local	Phase I	Calando Silence Therapeutics AG

Acute renal failure	Systemic	Phase I	Quark Pharmaceuticals/Pfizer

Diabetic macular edema	Topical	Phase II	Silence/Quark/Pfizer

Metastatic melanoma	Local/direct	Phase I	Duke University

Pachyonychia congenita	Topical	Phase Ia/b	Transderm

High cholesterol	Systemic	Phase I	Tekmira Pharmaceuticals Corporation

Asthma	Systemic	Phase II	ZaBeCor Pharmaceuticals

HIV	Direct	Phase I/II	Benitec/City of Hope

The first clinical trial conducted using siRNA was aimed at age related macular degeneration (AMD) [40]. As early as 2004, the company Sirna presented the first ever clinical data for an RNAi-based drug - the compound AGN-745, formerly Sirna-027 - against AMD [41]. The company OPOKO Health launched the first ever siRNA Phase III trial in 2007 using Bevasiranib, a first-in-class siRNA drug designed to silence the genes producing Vascular Endothelial Growth Factor (VEGF), believed to be largely responsible for vision loss in wet AMD. Unfortunately in March 2009, the phase III trial was terminated ahead of schedule due to a review by an independent data committee, which found that although the drug showed activity, the trial was unlikely to meet its primary endpoint (OPOKO Health, Miami, Florida, press release). One should keep in mind that the clinical trials were performed with unmodified siRNAs and no doubt showed good results, but apparently not convincing enough for human therapy.

Failure of this first clinical phase III study highlights the need for second generation siRNA therapeutics, for example shRNA-expression cassettes, as well as efficient transfer vehicles for these cassettes.

## Delivery of interfering RNA

In principal, there are many different ways to trigger RNAi. Most of the proposed clinical applications of RNAi incorporate chemically synthesized 21 nt siRNA duplexes with 2 nt 3' overhangs. This mode of administration is transient, since intracellular concentrations of the siRNAs are diluted during cell division. Furthermore, duplex siRNAs are negatively charged polymers and therefore do not easily penetrate hydrophobic cellular membranes without assisting carriers. In addition, unprotected and unmodified siRNAs are generally rapidly degraded by serum RNases.

In contrast, intracellularly expressed short hairpin RNAs mediate long-term knock-down of target transcripts for as long as the shRNA is transcribed. Therapy of chronic diseases, for example, requires exactly this - long-term target gene down-regulation. An ideal delivery vehicle should therefore facilitate endosomal/lysosomal escape and, in the case of shRNA, the payload must penetrate the nuclear membrane. Viruses and vectors derived from them carry out precisely these tasks and have therefore become a major delivery system for shRNA.

RNAi in cells can be induced from intracellularly expressed short hairpin RNAs either by shRNAs or synthetic miRNAs [2,42,43]. The basic transcriptional unit of shRNA is sense and antisense sequences connected by a loop of unpaired nucleotides. MiRNA stem loops are typically expressed as part of larger primary transcripts (pri-miRNAs) [44]. Artificial miRNAs more naturally resemble endogenous RNAi substrates and are more amenable to Pol-II transcription and may seem to be more attractive for therapies [44,45]. But to date shRNA- and artificial miRNA-based strategies have been compared with conflicting results [46-48] and it seems that the choice is always dependent on the strategy and the desired outcome and has to be figured out experimentally [49].

## Viral delivery of shRNA expression cassettes

Combining RNAi with viral gene therapy vectors is a powerful approach in certain scenarios where spatiotemporal control over gene silencing is highly critical, and/or where persistent suppression of a target is mandatory for success. The design of viral RNAi vectors became possible upon discovering that promoter-driven expression of short hairpin RNAs (shRNAs) induces the RNAi machinery [50]. This strategy involves cloning an oligonucleotide containing the siRNA sequence into plasmid or viral vectors to endogenously express shRNA, which is subsequently processed in the cytoplasm to siRNA. Fortunately, shRNA expression cassettes are extremely limited in size, meaning they can be packaged into even the smallest known viral vectors. The next section reviews existing viral vector systems.

### Adenovirus vectors

Adenoviruses (AdV) belong to the family of *Adenoviridae*, and adenoviral vectors are frequently used for experimental gene therapy and 25% of clinical gene therapy trials currently underway are using adenovirus [51]. Adenoviruses are medium-sized, non-enveloped viruses with a nucleocapsid and a linear dsDNA genome. They are able to replicate in the nucleus of mammalian cells but do not efficiently integrate into the host's genome. AdVs are able to package approximately 8-30 kb of foreign DNA. Several AdV features are attractive for vector use, including infection of both dividing and non-dividing cells, high levels of transgene expression and the ability to grow to high titers *in vitro*. There are 53 described serotypes in humans, and AdVs are responsible for 5-10% of upper respiratory infections in children and many infections in adults. Hence seropositivity to AdV is frequently observed, a drawback for gene therapy using AdV. Entry of adenoviruses and their vectors into cells involves two sets of interactions between the virus and the host cell. First, the viral fiber protein binds to the cell receptor, either CD46 for group B human adenovirus serotypes or the coxsackievirus adenovirus receptor (CAR) for all other serotypes. The initial binding is followed by a secondary interaction, where the penton base protein interacts with an integrin, resulting in entry of virions into the host cell [52]. Adenoviral vectors exhibit no clear tissue tropism, however the relevant surface receptors are often absent in the tissue of interest (especially in tumor cells).

In connection with RNAi therapy the large packaging capacity of a gutless adenovirus vector could be a problem for small shRNA cassettes, since they might jeopardize genetic vector stability [53]. A further disadvantage of AdV vectors is the problem of repeated administration, which can trigger a strong immune response, potentially limiting their effectiveness in certain therapeutic settings. Additionally, the frequently described liver toxicity [54,55] makes adenoviral vectors unsuitable, or at least to be handled with care in human therapy.

Today, adenoviral vectors are a common delivery method to introduce shRNA-expression cassettes into target cells *in vitro *and are commercially available. Several publications report the use of adenoviral vectors for transducing RNAi-based therapies *in vivo*. The first study employing an adenoviral vector for *in vivo *RNAi was published in 2002 for an application in the central nervous system [42]. These data validate the outstanding promise of oncolytic adenoviral vectors for tumor-restricted shRNA expression [56], although still with the limitations mentioned above. Different reviews have summarized adenoviral shRNA delivery in great detail (e.g. [57]) and the interested reader should refer to them.

### Adeno-associated virus vectors

Adeno-associated viral (AAV) vectors have also been tested in clinical studies in multiple tissues [58]. AAV is one of the smallest viruses and belongs to the genus *Dependovirus *and the family *Parvoviridae*. It has a small, single-stranded DNA genome (4.8 kb) and is apathogenic in humans (at least according to current knowledge). The genome contains only two genes, which can be replaced with foreign ones, leaving only the terminal ITRs to allow high-level expression of the insert. However, the 5 kb packaging limit of AAV is still sufficient to accommodate at least eight individual shRNA expression cassettes [19,59]. The virus is replication-defective and until recently required adenovirus for replication and production of vectors. New methods for producing recombinant AAV using single adenoviral genes have made adenoviral co-infection of AAV-producing cells dispensable [60]. Although wild-type AAV preferentially integrates within a specific region of human chromosome 19, recombinant AAV is engineered to be inefficient in integration since it lacks the AAV Rep protein [61,62].

In contrast to adenovirus, pseudotyping of AAV permits entry retargeting, allowing delivery of the shRNA cassette to specific cells or tissues [63]. Furthermore, AAV-vectors show only low reactivity with cellular immune responses.

To date, several reports have already described the development of AAV vectors delivering and expressing anti-tumor shRNAs *in vitro *as well as in small animal models. One study exploited AAV expressing shRNAs against Hec1 (highly expressed in cancer 1) [64]. Repeated intratumoral administration caused anti-proliferative and pro-apoptotic effects in tumor cells. Another recent study dealt with shRNA mediated down-regulation of the androgen receptor (AR). Systemic delivery of recombinant AAV vectors stably expressing shRNA against the AR gene eliminated prostate xenografts in nude mice [65]. Details and further possible applications for AAV in shRNA delivery can be found elsewhere (e.g. [66,67]).

Both adenoviral (AdV) and adeno-associated virus (AAV) vectors are non-integrating and therefore pose only minimal risks of insertional mutagenesis. At the same time, this may represent a negative aspect since the genetic information is less stably conserved and may be lost during repetitive cell division. So, although these vectors transduce both dividing and non-dividing cells allowing very efficient gene transfer, they are inadequate for long-term gene replacement therapy.

### Retrovirus vectors

The use of gene delivery vectors based on retroviruses was introduced in the early 1980 s by Mann *et al. *[68]. These single-stranded (ss)RNA viruses belong to the family of *Retroviridae *and replicate through a double-stranded DNA intermediate. They integrate their genomes stably into the host cell DNA allowing long-term expression of inserted therapeutic genes. The subfamily of *Orthoretrovirinae *comprises different genii, for example the simplest Gammaretroviruses (e.g. MLV) and the more complex Lentiviruses (e.g. HIV). The viral genome is approximately 10 kb, containing at least three genes: *gag *(coding for core proteins), *pol *(coding for reverse transcriptase) and *env *(coding for the viral envelope protein). Complex retroviruses encode a number of accessory proteins that are involved in regulating viral replication or the host cell response to the virus. At each end of the genome, long terminal repeats (LTRs) contain promoter/enhancer regions and sequences involved in integration. In addition there are sequences required for packaging the viral RNA (psi Ψ^+^).

Retroviral entry and genome integration do not require viral protein synthesis; therefore all viral genes in the vector genome can be replaced with foreign sequences. Vector particles are produced by packaging cell lines that provide the viral proteins *in trans*. These cells release vector genomes packaged into infectious particles that are free from contaminating helper virus and replication-competent recombinant virus. Hence, no viral proteins are produced after transduction, avoiding inducing adverse effects or immune responses against the vector particle, and preventing subsequent spread of the vector.

When exiting the cell, retroviruses and their vectors acquire cell-derived lipid bilayers containing inserted glycoproteins (Env) by budding from the host cell membrane. The Env protein mediates attachment and fusion between the next host cell membrane and viral membrane, which results in release of the viral capsid particle containing the genetic material into the cytoplasm. This plays a central role in targeting retroviral entry to target cells, since Env interacts with a specific cellular protein and accordingly determines viral tropism.

Altering the *env *gene or its product is one possible way to manipulate the target cell range [69-72] and increase the vector's safety. The most successful approach to enhancing safety for tumor therapy is engineering protease-activated Env proteins. In this system, viruses remain non-infectious until Env becomes activated via cleavage by a secreted or membrane-bound protease that recognizes an engineered protease substrate [73,74]. More detailed information can be found elsewhere [75-78]. Selective infection of tumor cells combined with transfer of anti-tumoral sh/mi/siRNAs is an attractive strategy for cancer therapy, and is the focus of current research. Several strategies have been explored, and summaries can be found elsewhere [79].

The use of retroviral vectors for efficiently introducing shRNA expression cassettes into target cells has been exploited for many years now. Retroviruses were among the first vectors used as transfer vehicles for hairpin-RNA expressing plasmids. Brummelcamp *et al. *[50] used retroviruses and highlighted the extreme specificity of the RNAi concept, fanning interest in using RNAi for therapeutic applications and cancer therapy. A number of publications followed, using retroviral vectors based on Murine Leukemia Virus (MLV) as transfer vehicles for shRNA-expression cassettes. The most prominent were the works of Paddison *et al. *[80] and Berns *et al. *[81] both published in 2004. The groups generated retrovirus-based shRNA expression libraries capable of targeting around a third of all human genes. These libraries showed promise in gene analysis and discovery since they enabled large-scale genetic screens and offered a tool for identifying genes involved in specific biological processes.

Other work followed [48,82,83] applying retrovirally delivered shRNA for high throughput screening. More detailed views on pioneering experiments can be found elsewhere (e.g. [18]), and retroviruses are still currently being used as transfer vehicles for shRNA [84,85].

One recent study reported prolonged suppression of productive HIV-1 infection in a T-cell line (Molt-4) by a retrovirally (MLV) delivered shRNA targeting a sequence located within the NF-κB binding motif of the HIV-1 promoter. HIV-1 expression in the shRNA expressing CD4(+) T-cell line was suppressed for 1 year [86].

### Lentivirus vectors

Lentiviruses (LV) constitute a subclass of retroviruses and also carry two copies of a single-stranded RNA genome in an enveloped capsid. Among the different species in the genus *lentivirinae*, the most prominent is the Human Immunodeficiency Virus (HIV), as well as others such as the Feline Immunodeficiency Virus (FIV) or Simian Immunodeficiency Virus (SIV).

In contrast to the retroviral vectors above mentioned, lentiviral vectors are capable of transducing dividing and non-dividing cells (e.g. neurons), which makes them preferred candidate vectors for nervous system applications. Lentiviral vectors can accommodate large (up to 7.5 kb) amounts of DNA [87], are less immunogenic than adenoviral vectors and are mostly used for local applications as well as for *ex vivo *gene therapy. They are more complex than simple retroviruses, containing additional six proteins, *tat*, *rev*, *vpr*, *vpu*, *nef *and *vif*. Since the native viruses can cause fatal diseases in humans, non-replicating viruses are used for transgene expression. Current packaging cells are usually transfected with separate plasmids encoding for an *env *gene, a transgene construct and a packaging construct supplying the structural and regulatory genes *in trans *[88].

The most advanced and safest forms are the engineered "self-inactivating" (SIN) vectors. Here, the U3 region of the UTR is deleted, and a heterologous promoter (such as CMV) ensures transcription of the entire vector mRNA. This strategy excludes any risk of recreating replication competent wildtype-like viruses by chance.

Just like the gamma-retroviruses, lentiviral vectors are amenable to pseudotyping. For example, pseudotyping of *env *with VSV-G broadens tropism and supports uptake into otherwise refractory cells, such as human hematopoietic or embryonic stem cells [89].

Lentiviruses are commonly used as vectors for the transfer of shRNA-expression cassettes. Today, delivery of shRNAs into target cells via lentiviral vectors is so efficient that various companies offer this method for *in vitro *experiments. During the past 10 years, several adaptations and novel techniques have emerged to improve (conditional) transgene expression, and the assiduous scientist can choose between different well-established systems for their experimental setup.

Examples for the use of lentiviral vectors as vector systems for shRNA are innumerable, so here, we will only touch on a few. In many cases, lentiviral vectors have been employed successfully to regulate target genes in the brain after local injection [42,90,91]. In an upcoming clinical trial one application of RNAi will involve *ex vivo *lentiviral vector delivery of an shRNA expression cassette into hematopoietic stem cells collected from patients infected with HIV. The transduced cells must be re-infused into these patients for a therapeutic benefit *in vivo *[92]. LV have also been used to create transgenic animals, but one drawback is the fact that vectors become silenced after long-term culture [93]. Different reviews give exhaustive surveys in great detail, e.g. [94-97].

### Baculovirus

The insect baculovirus is in its very early testing stages as a possible vector for *in vivo *use and as vector for shRNA [98]. Reports on baculovirus-delivered shRNA comprise manageable amounts of publications. Vectors based on baculovirus can transport large amounts of genetic data leaving copious space for creative combination of gene therapy and silencing vectors [99]. Furthermore, baculovirus is unable to replicate and express viral proteins in mammalian cells, making the virus a safe gene therapy candidate now in its first developmental stages. Baculovirus-based shRNA expression is currently used to target different viral infections, for example HCV replication [100,101] as well as Influenza virus A and B [102]. However, the effects are transient, since a major limitation of baculoviral transduction vectors is the short duration of transgene expression. There are ways to overcome this, for example by inserting Epstein-Barr Virus sequences into the baculovirus vector to improve long-term expression [103]. There is still a long way to go before the promising results find their way into human therapy trials.

### Replicating Viruses

Effective gene-based therapies not only require efficient delivery of therapeutic genes to targeted mammalian cells but also continuous gene expression. Now, the realization that conventional gene therapy approaches have yet to deliver significant therapeutic benefit for cancer treatment, combined with advances over the past 25 years, has re-ignited interest in using replicating viruses. The big advantage of replicating viruses in contrast to replication-defective vectors is that they are able to spread through tumor tissue by viral propagation. In this setting, each transduced/infected tumor cell becomes a virus-producing cell, thereby sustaining further infection beyond the initial inoculum. This idea led to a novel cancer therapy: oncolytic virotherapy. Recent advances in molecular biology have allowed the design of several genetically modified viruses, such as Adenovirus [104,105] and Herpes Simplex Virus [106-108] that specifically replicate in and kill tumor cells. Also Reovirus [109,110], Poliovirus [111,112], Paramyxovirus [113], Vaccinia Virus [114-117] and Vesticular Stomatitis Virus [118] are being exploited. These viruses possess intrinsic oncolytic activity since infection finally leads to host cell death. In contrast, LV and MLV have no oncolytic activity, and shRNA-expression cassettes are used to fulfill effector functions.

### Conditionally replicating lentiviruses

Fully replicating lentiviruses such as HIV, due to their calamitous risk to benefit ratios in healthy patients, are not up for discussion as gene transfer vehicles. However, in already infected HIV-1-positive patients gene transfer using conditionally replicating lentiviral vectors are under consideration [119,120]. A strategy based on exploiting an HIV-based lentiviral vector carrying an antisense sequence targeting HIV to treat HIV infection has entered clinical trials. This trial is evaluating a conditionally replicating HIV-1-derived vector pseudotyped with VSV-G expressing an 937-base antisense gene against the HIV envelope [120]. The novel idea is retaining the full HIV LTRs in the vector, resulting in upregulated expression of the antisense upon wildtype HIV infection of the cell. The study showed improved cellular responses to HIV in four out of five subjects, and three experienced improvement in their T cell memory responses.

As opposed to the antisense-strategy used above, another group is studying shRNA-expression cassettes as transgenes in replicating HIV-1, using a doxycyclin-dependent HIV-1 variant [119]. The virus replicated conditionally in the presence of doxycyclin (dox) and efficiently delivered anti-nef shRNAs to those cells susceptible to HIV-1 infection. Dox withdrawal generated cells containing a silently integrated provirus with an active shRNA expression cassette. Removal of nef-sequences from the vector genome avoided vector self-targeting but inhibited HIV-1 replication in transduced cells *in vitro*.

The use of (conditionally) replicating lentiviral vectors seems promising in the treatment of HIV-1 infections using shRNA and may prove beneficial for this therapy spectrum.

### Replication competent MLV

Unique among the replicating viruses being developed as oncolytic agents, retroviruses, in particular MLV-based viruses, replicate without immediate lysis of host cells and can maintain viral persistence through stable integration.

Retroviruses have been studied extensively for almost 100 years and the work until now has culminated in the first clinical trials [121] using replicating MLVs *in vivo*. MLV exhibits tumor selectivity due to its inability to infect quiescent cells and can achieve highly selective and stable gene transfer throughout entire solid tumors *in vivo *at efficiencies of up to >99% even after initial inoculation at MOIs as low as 0.01 [122]. One obstacle to overcome in tumor therapy using replication competent retroviruses is the restricted size for inserted transgenes. The size of the MLV genome is limited to roughly 11 kb and viral genes cannot be replaced by transgenes.

One possibility to overcome this restriction is engineering MLVs in a semi-replicative setting. The idea is to split the viral genome onto two transcomplementing vectors, each carrying the genetic information for either *gag/pol *or *env *and/or a transgene. Only together - not alone - these vectors contain the genetic material necessary for replication and vector production upon cell transfection. We and others developed this idea [71,123,124], where the *gag*/*pol *and *env *genes are split between two viral genomes. The duo allows co-propagation of two different transgenes, which offers both a back-up therapeutic opportunity, should the effect of the first gene product wane due to developing drug resistance, and a means for vector replication shut off, if the transgene is a suicide gene. The split genomes also enhance the capacity for inserting a therapeutic gene. We constructed split viral genomes and used fluorescent proteins to visually monitor viral replication of the resulting SRRVs [71].

Replication competent viruses containing the complete genome can also be used to carry transgenes. In 2001, Logg and Kasahara [125] conducted studies testing the insertion capacity of replicating viruses and found that MLVs containing inserts of 1.15 to 1.30 kb replicated with only slightly attenuated kinetics compared to wild-type and efficiently spread transgenes after *in vivo *administration.

Many reports describe replicating MLVs for gene therapy using inserted suicide genes [126]. We pioneered replicating MLVs as transfer vehicles for shRNAs to amplify shRNA delivery [127]. Our replicating MLV constructs express shRNA under the control of an RNA pol III promoter [50,128]. Inserting the cassette did not interfere significantly with viral fitness, and vectors were genetically stable and functional in silencing target gene expression. Our results show that replicating MLVs are excellent tools for efficient delivery and expression of shRNAs, have great potential for functional genomics, and might be suitable for *in vivo *cancer gene therapy, if combined with efficient entry targeting. We are currently focusing on *in vivo *studies using these viruses and look forward to promising results.

## Conclusions

RNAi technology has become a powerful tool and key method for gene therapy in the scientist's hands. Although the effectiveness of RNAi is undoubted, there are still limitations to exploiting the technology properly due to inefficient delivery and distribution of the shRNA-cassettes into the target cells. Focusing on viral delivery of shRNA, we highlighted that viruses and vectors derived from them are excellent candidates to deliver shRNA into the desired tissue or cells. We discussed different methods for viral delivery of shRNA expression cassettes using conventional methods and revealed promising new strategies utilizing replicating retroviruses.

## Competing interests

The authors declare that they have no competing interests.

## Authors' contributions

KS and BS contributed equally to conception, design and acquisition of data. Both have been involved in drafting the manuscript and give final approval of the version to be published.
